# Cellulose-Based Metallogels—Part 1: Raw Materials and Preparation

**DOI:** 10.3390/gels9050390

**Published:** 2023-05-08

**Authors:** Aleksandra Mikhailidi, Irina Volf, Dan Belosinschi, Bogdan-Marian Tofanica, Elena Ungureanu

**Affiliations:** 1 Higher School of Printing and Media Technologies, St. Petersburg State University of Industrial Technologies and Design, 191186 St. Petersburg, Russia; 2 Faculty of Chemical Engineering and Environmental Protection, “Gheorghe Asachi” Technical University of Iasi, 73 Prof. Dr. Docent D. Mangeron Boulevard, 700050 Iasi, Romania; 3 Département de Chimie-Biologie/Biologie Medicale, Université du Québec à Trois-Rivières, Trois-Rivieres, QC G8Z 4M3, Canada; 4 IF2000 Academic Foundation, 73 Prof. Dr. Docent D. Mangeron Boulevard, 700050 Iasi, Romania; 5 Department of Exact Sciences, “Ion Ionescu de la Brad” University of Life Sciences Iasi, 3 Mihail Sadoveanu Alley, 700490 Iasi, Romania

**Keywords:** cellulose, metallogels, hydrogels, cellulose-based gels, dissolution

## Abstract

Metallogels are a class of materials produced by the complexation of polymer gels with metal ions that can form coordination bonds with the functional groups of the gel. Hydrogels with metal phases attract special attention due to the numerous possibilities for functionalization. Cellulose is preferable for the production of hydrogels from economic, ecological, physical, chemical, and biological points of view since it is inexpensive, renewable, versatile, non-toxic, reveals high mechanical and thermal stability, has a porous structure, an imposing number of reactive OH groups, and good biocompatibility. Due to the poor solubility of natural cellulose, the hydrogels are commonly produced from cellulose derivatives that require multiple chemical manipulations. However, there is a number of techniques of hydrogel preparation via dissolution and regeneration of non-derivatized cellulose of various origins. Thus, hydrogels can be produced from plant-derived cellulose, lignocellulose and cellulose wastes, including agricultural, food and paper wastes. The advantages and limitations of using solvents are discussed in this review with regard to the possibility of industrial scaling up. Metallogels are often formed on the basis of ready-made hydrogels, which is why the choice of an adequate solvent is important for obtaining desirable results. The methods of the preparation of cellulose metallogels with d-transition metals in the present state of the art are reviewed.

## 1. Introduction

Naturally derived gels are emerging materials that display specific physical and chemical properties that enable their use in a wide variety of applications [1].

Generally, hydrogels can be prepared from synthetic or bio-based polymers. Synthetic polymers are the products of petroleum, while bio-based polymers have natural sources—they are derived from plants and living organisms [2,3,4,5]. Hydrogels from synthetic polymers are less hydrophilic and mechanically more robust compared to bio-based hydrogels [6]. However, the latter are far more preferable due to their unique advantages, such as non-toxicity, biodegradability, low cost, renewability and abundance of the sources [1,7,8,9].

Cellulose, starch, chitin, and chitosan are examples of natural-based biopolymers with hydrophilic functional groups that can absorb and retain a large amount of water [7,8,9,10,11,12]. Cellulose is a pristine material for several thousand types of useful products for the chemical industry, including oligo- and monosaccharides and their derivatives, fibers, films and other materials [8,13,14]. Cellulose is preferable for the production of hydrogels and their further functionalization from economical, ecological, physical, chemical, and biological points of view since it is inexpensive, renewable, versatile, non-toxic, reveals high mechanical and thermal stability, has a porous structure, an imposing number of reactive OH groups, and good biocompatibility.

Hydrogels are mostly produced from nanocellulose [15,16] and bacterial cellulose [17,18], both are not advantageous at an industrial scale, while plant-derived cellulose in the current state of the art has comparatively less often been applied in the preparation of hydrogels because of its poor solubility. This drawback can be overcome by obtaining cellulose derivatives via various chemical modification procedures, such as esterification, etherification, or oxidation [7,15,19,20,21,22]. However, this means additional steps of derivatization and different properties of the derivatives because of the introduction of the new functional groups.

Non-derivatized cellulose hydrogel can be obtained from a cellulose solution through physical cross-linking by hydrogen bonding with hydroxyl groups [23]. In this case, the three-dimensional polymer structure captures the solvent molecules in the framework cavities due to various supramolecular interactions, namely hydrogen bonding, π–π stacking, or van der Waals interactions [24]. Additionally, a number of cellulose hydrogels preparation methods include chemical cross-linking in order to improve the properties of the hydrogel. Epichlorohydrin (ECH), aldehydes and aldehyde-based reagents, urea derivatives, carbodiimides and multifunctional carboxylic acids are the most widely used crosslinkers for cellulose [25]. 

Cellulose-based hydrogels give rise to a huge number of composite materials. They possess three-dimensional porous structures, which allow the incorporation of functional fillers and are thus applied as smart materials [16,22,26,27,28,29,30]. The same, the use of cellulose aerogels is preferable to obtain special properties with new potential applications [27,28,29,30,31]. Nanocomposites based on cellulose hydrogels with metal particles have been extensively developed in recent decades. In such compounds, cellulose acts as a matrix for metallic and metal oxides nanostructures. These metal/cellulose composites could be synthesized via chemical (atomic or molecular condensation, sol-gel process, chemical vapor deposition, colloidal method), physical (ultrasonication, laser pyrolysis or ablation, spluttering and others) or biological organisms (e.g., bacteria, fungi, viruses, algae, plants act as reducing or stabilizing agents) routes [31]. 

The most desirable for biomedical applications are small nanoparticles (less than 100 nm) uniformly distributed over the bulk of the hydrogel [29]. Although for some purposes, e.g., wound dressings, the presence of nanoparticles only in the surface layers may be sufficient. Therefore, the methods of nanoparticle synthesis are focused on the possibility of managing the size and distribution of the particles due to the selection of special conditions for the method [32,33,34,35]. Additionally, since metal nanoparticles tend to agglomerate and, as a result, grow and reach bigger sizes (up to dozens of microns), special reagents—stabilizers gain significant importance. The matrix for the introduction of nanoparticles, a cellulose hydrogel, plays an important role too. It traps the particles and holds them due to the physical and chemical bonding performing as the additional stabilizer of growth and prevents washing the particles out. Thus, metal particles, penetrating into the polymer matrix and coordinating with the functional groups of the cellulose hydrogel, form a metallogel in which the metal is a part of the gel network as a coordinated metal ion (in a discrete coordination complex), as a cross-linking metal node with a multitopic ligand (in coordination polymer), and as metal nanoparticles adhered to the gel network [24]. These materials are called metallogels [24], or gel-nanocomposites [36], or metal nanocomposites [37]. However, the latter is a much broader term, as includes a wide range of materials other than polymer gels. 

There is a significant number of review articles of recent years which consider cellulose hydrogels and their applications [1,2,7,8,15,18,26,31,36,38,39,40,41,42]. This fact perfectly reflects the tremendous interest of researchers and industry in natural polymer hydrogels. Listed critical reviews are focused on the classification of hydrogels, production issues and especially on the applications of hydrogels from cellulose and its derivatives. Antimicrobial cellulose hydrogel with embedded silver nanoparticles is probably the most common and usually the only example of an application of a cellulose–metal composite material in the field of biomedicine [26,36,38,39] and food packaging [8,19], while the other cellulose metallogels are rarely discussed in the reviews on cellulose hydrogels.

On the other hand, there are probably even more review articles on the topic of polymer composite materials with metal nanoparticles [43,44,45,46,47,48,49,50], including reviews about composite materials based on cellulose [48,49,50]. Though, the paper provided by Anžlovar and Žagar [48] discusses only a few examples of pristine cellulose hydrogel/metal composites, Oprea and Panaitescu [49] consider just nanocellulose hybrids with different metal oxides, and Abdellatif et al. [50] collects the data about cellulose derivatives. Therefore, the questions connected to pristine cellulose metallogels have not been addressed yet. The given critical review is designed to focus on the production of the non-derivatized cellulose hydrogels and metallogels derived from them.

## 2. Cellulose Metallogels

At present, composite materials are of great interest. Metal nanoparticles incorporated into the polymer matrix add new properties to the composite, expanding the scope of its application, whereas the amount of metal can be rather small or, on the contrary, it can make up a big share of the composite. The need for control of the particle dispersiveness, their size, and organization on the surface and in the bulk of polymer supports is one of the main tasks. 

The first approach to the fabrication of cellulose metallogels is to prepare metal nanoparticles separately and then mix them with the polymer; then cross-linking takes place after mixing the polymer solution and the suspension with nanoparticles [51,52]. This method leads to a uniform distribution of nanoparticles in the polymer matrix; however, it is a multistaged procedure, and it involves expensive industrially produced nanoparticles. The second, single-pot and cost-effective approach to obtaining metal nanoparticles is the reduction of metal ions from the aqueous solutions of their salts directly inside the polymer matrix [53,54,55]. This process is known as the diffusion-reduction method and it is available to all types of cellulose matrices, including hydrogels and metallogels. Cellulose suits the production of metallogels due to its extensive pore network. The pores limit the agglomeration of metal nanoparticles and promote their fixation both on the surface and in the inner layers of the cellulose material. It was shown that the structure of cellulose after the introduction and reduction of metal nanoparticles did not change. Therefore, cellulose is considered a neutral nanoreactor [53]. Hydrogels produced from pristine cellulose along with aerogels, films, powder samples, and fibers were successfully applied as scaffolds (carriers, matrices, sacrificing templates) for intercalation of transition zero-valent d-metals, such as cobalt, nickel, silver, gold, platinum, palladium, iron, copper, zinc or/and their oxides [51,52,53,54,55,56,57,58,59,60]. For example, it was shown that the hydrate cellulose film could be successfully used to obtain nickel nano- and microspecies located mainly on the surface or in the near-surface layer. A specific feature of nickel species synthesis and embedding into the cellulose film was that the reduction and formation of nickel particles occurred in the solid matrix, which somehow limited the growth of species [53]. In another study, nanosized cobalt particles were prepared by chemical reduction within a microcrystalline cellulose matrix. Two different chemical reducers, NaBH_4_ and NaH_2_PO_2_, were applied. Particles obtained via the NaBH_4_ reduction were amorphous Co-B or CoO composites with diminished ferromagnetic behavior and particles made via the NaH_2_PO_2_ reduction were well-ordered ferromagnetic cobalt nanocrystals [55]. The synthesis of the nanoparticles was carried out under heterogeneous conditions which helped to limit the agglomeration of metal particles. The same occurs in fibers or hydrogels, for instance, the silver and gold nanoparticles were embedded into the cellulose matrices in [56]. Composites cellulose–Au and cellulose–Ag contained a low amount of reduced metals and demonstrated antimicrobial properties. All these examples used the diffusion-reduction method to obtain hybrid cellulose composites with metals. This way may be successfully adapted to the preparation of metallogels via modification of the ready hydrogels.

The first step of the diffusion-reduction process is immersing the prepared hydrogel into the aqueous solution of metal salt to provide diffusion of the metal ions into the cellulose matrix. The size distribution of the nanoparticles can be controlled by varying the concentrations of the salts. The reducer is added in the second stage of the process. The reaction results in the colloidal suspension outside the hydrogel, while inside it the metal ions transform to zero-valent metal particles. Finally, the cellulose metallogels are washed with distilled water to remove unfixed metal particles [54,56]. 

**Figure 1:**
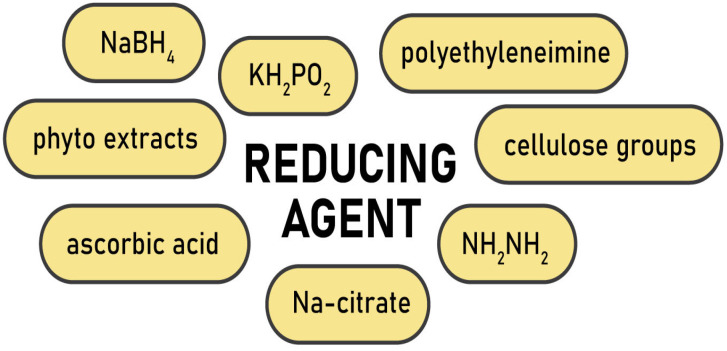
Reducing agents for production of metal nanoparticles.

**Figure 2:**
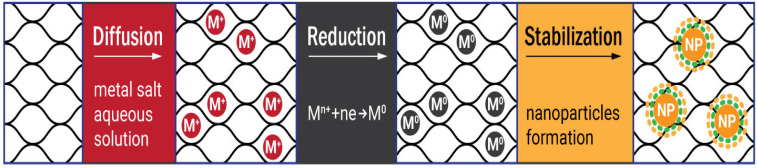
The scheme of the diffusion-reduction Turkevich process in the bulk and on the surface of the hydrogel matrix.

Depending on the type of metal and the desired characteristics of the nanoparticles, different reducing agents are used (Figure 1). For instance, active sodium tetrahydridoborate or mildly active potassium hypophosphite and hydrazinium hydrogen sulfate are often applied as reducers for zero-valent metals such as Ni, Co, Cu, Fe [37,53,61], and sometimes for Au [62] and Ag [63]. Ascorbic acid was employed to prepare cellulose–Ag nanocomposites in the microwave-assisted process [64], while polyethyleneimine reduced Au^+^ from an aqueous suspension [65]. However, for noble metals a relatively simple and reproducible Turkevich method is preferable. It engages trisodium 2-hydroxypropane-1,2,3-tricarboxylate (trisodium citrate) to reduce silver and gold ions for the synthesis of spherical nanoparticles (Figure 2) [54,56,66]. 

**Figure 3:**
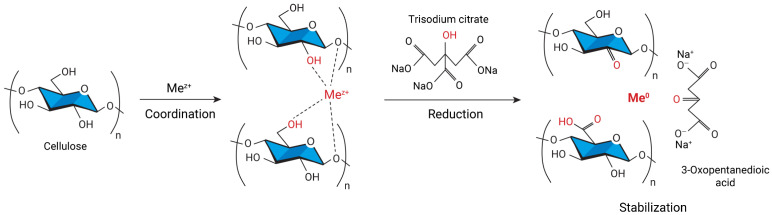
The mechanism of the redox reaction leading to the formation of metal nanoparticles in the cellulose matrix.

The mechanism of metal reduction by the Turkevich method is shown in Figure 3. Metal ions diffuse into the cellulose matrix and coordinate with its functional groups. The reducing agent, trisodium citrate, is oxidized in this reaction to 3-oxopentanedioic acid, some of the cellulose OH groups are oxidized to carbonyl or carboxyl groups, and the metal ions are reduced to the zero-valent state. The metal particles grow and form a nanoparticle, which is stabilized by cellulose, 3-oxopentanedioic acid, and trisodium citrate. Stabilization of the particles prevents further agglomeration, which makes it possible to obtain smaller nanoparticles [35,56,67]. The metal reduction depth depends on the aspect ratio between cellulose and metal ions. The less the aspect ratio, the less the share of cellulose and, respectively, higher the amount of zero-valent metal in the solutions [56].

Strange as it may sound, the reduction step may be carried out without reducers due to the functional groups and bounds of the cellulose matrix of the hydrogel. In this case, it is usually called “green synthesis”. Cellulose contains the end aldehyde groups; therefore, the reaction occurs without the addition of chemical reducers. For example, Au(0) nanoparticles were synthesized in a cellulose hydrogel without a reducer in [56]. However, cellulose has a rather moderate number of end aldehyde groups, not enough to interact with a big number of metal ions. As a result, only low content of metal nanoparticles may be obtained in the composite hydrogels. Cellulose derivatives are able to perform the reduction of metal ions as well. For instance, Ag nanoparticles were obtained in a carboxymethyl cellulose (CMC) hydrogel, where the reducing agent was CMC [68]. Not only cellulose functional groups but also cross-linking bounds in the polymer can provide reduction, for instance, C = N bonds in the hydrogel prepared from amino cellulose and dialdehyde xylan [16]. 

Another “green” option for obtaining metal nanoparticles by reduction from their salts but without chemicals is a phyto-synthetic route which is related to the use of aqueous plant extracts. Plant parts (leaf, stem, root, fruit, and seed) produce phyto-chemicals naturally rich in amino, carboxyl and hydroxyl groups for triggering the formation of metal nanoparticles, as well as stabilizing and/or capping them [69]. Phyto-synthesis has a good scaling potential for the production of stable, varied in shape and size nanoparticles of metals and metal oxides. Currently, this biosynthetic way is commonly used for the production of ZnO nanoparticles from plants such *as Ferulago angulata*, *Bergenia ciliata*, *Sageretia thea*, *Anisochilus carnosus*, *Limonia acidissima* L., and *Syzygium cumini* [69,70,71,72,73,74]. 

Metals can also initiate the gelation process and act as cross-linking agents during the formation of metallogels. The ability of transition monovalent metal ions to start the gelation of nanofibrillated cellulose (NFC) was revealed by Dong and his research group. During the TEMPO process that was applied for NFC production, negatively charged surface carboxylate groups that provide high binding capability to transition metal species (e.g., Ag^+^) were generated. Ag^+^ was bound on the NFC surface and simultaneously induced the formation of NFC-Ag^+^ hydrogels. Subsequently, Ag^+^ ions were slowly reduced to Ag(0) nanoparticles by hydroxyl groups on NFC without an additional reducing agent. Thus, the stiff NFC-Ag^+^ hydrogel was initiated by strong association of carboxylate groups on NFC with Ag^+^ and sufficient NFC surface charge reduction [75]. Zander and co-workers reported novel nanocellulose hydrogels fabricated via hydrogelation using metal salts. In this method, Ca^2+^ and Fe^3+^ played the role of cross-linkers [76].

In contrast to metallic nanostructures that are mainly limited to noble or semi-noble metals, practically any type of metal oxide can be deposited on the cellulose surface with TiO_2_, Fe_3_O_4_, and ZnO being the most abundant. For this purpose, various cellulose types have been applied, spanning from natural cellulose fibers to cellulose nanocrystals in the original or chemically modified form [48].

Summing up, we can highlight two main chemical methods for the preparation of cellulose-based metallogels. The first one is a modification of the diffusion-reduction method, when the hydrogels prepared in advance are immersed in a solution of a salt, and then the metal ions react with chemical reducing agents resulting in the metal nanoparticles. Additionally, the reduction may be carried out due to the reducing ability of cellulose itself or phyto-chemicals. This method can be utilized to obtain metallogels with almost any transition metal, among which silver is the most popular. The second method involves the direct participation of metal ions in gelation or cross-linking after mixing a solution of salt with a cellulose solution.

## 3. Raw Materials

As it was mentioned above, one of the most significant reasons for the utilization of natural polymers as raw materials for hydrogels is the fast renewability of the sources of bio-based compounds. Thus, usually cellulose is extracted from coniferous and deciduous wood which have the longest duration of renewal period among other cellulose sources—dozens of years, or from annual plants (renewal time is less than one year), seaweed (weeks for some species) and bacteria (days or hours). One more cellulose source is not a plant or living organism, but production and consumption waste. For wastes, the “period of renewal” is not even hours, but seconds, since the generation of waste throughout the globe in modern civilization is growing enormously. 

Cellulose for hydrogel production may be extracted from paper, food and agricultural wastes [77]. Rapeseed stalks [78,79,80], corn stalks [81,82,83], wheat straw [84], bagasse [85], flax fibers [86], thanaka heartwood [87], okara [88], tea leaf residues [89], sago pith [90], various fruit peel [91] and rinds [92], soybean stem [93], waste paper and cardboard [94,95,96,97,98] contain a sufficient amount of cellulose (Table 1) to extract and produce new functional materials, such as hydrogels. Thus, in agricultural wastes, such as annual plant residues, the content of cellulose varies in a wide range from 20 to 42% (Table 1) depending on the origin of the plant and the method of extraction. The lowest content of cellulose, 8.8%, has been shown for okara—a by-product of soymilk and tofu preparation. However, even with this small number, Cui and co-workers consider the production of cellulose hydrogels from okara to be inexpensive and eco-friendly [88]. The waste paper contains the biggest share of cellulose, but it has a heterogeneous composition, a share of impurities and additional components of papermaking.

Extraction of cellulose from agricultural wastes starts with washing, cutting and drying the parts of the plants. Then, the raw material is undergone acid or alkali hydrolysis at high temperatures for several hours. The final treatment is delignification or bleaching with sodium chlorite or hypochlorite, chlorine dioxide, hydrogen peroxide, peracetic acid, and others, using one of these chemicals or their combination [78,87,89,90]. Other sources of cellulose may require additional extraction steps. For example, okara needs to be further treated with surfactants after alkaline hydrolysis to remove proteins [8]. Usually, waste paper does not require hydrolysis, but it can be pre-treated with glacial acid to remove contaminants before dissolving, or activated by thermal defibration in hot water or alkaline solution, or the solvent replacement [93,95]. However, there is another example when the waste paper was pretreated with alkali hydrolysis to swell the cellulose fibers and remove residual ink particles and hemicellulose, then with mild acid hydrolysis to remove lignin residues, and finally, it was bleached in hydrogen peroxide and sodium hydroxide [97].

## 4. Preparation of Cellulose Hydrogels

As it was described above, metallogels are often produced from ready-made cellulose hydrogels by introducing a metal phase during a diffusion-reduction process or by mixing ready nanoparticles with the polymer. In both cases, the cellulose hydrogel must be prepared in advance by a researcher, since there is no industrial production of cellulose hydrogels yet. Seeing the choice of the fabrication method is quite challenging, we will discuss the most promising among them, comparing the advantages and disadvantages of various solvents.

One of the most common and easy routes to prepare cellulose hydrogels is the dissolution of cellulose or its derivatives in different solvents: organic, inorganic, mixed, and ionic liquids, followed by gelation and solvent removal [2,38,100]. There is no ideal resolution suitable for all purposes, every solvent has its own advantages and drawbacks. For instance, ionic liquids are expensive for use at an industrial scale (50 g of 1-allyl-3-methylimidazolium chloride costs from GBP 200 (available online: www.fishersci.co.uk, accessed on 29 January 2023) and demand special conditions, such as proper dehydration. These limitations in the present state of the art make the use of ionic liquids impossible to scale up, therefore, we will not discuss the manufacturing of cellulose hydrogels via dissolution in the ionic liquids in this article. Inorganic solvents, e.g., NaOH, NaOH/urea, or NaOH/thiourea/urea aqueous systems, are way cheaper but require cross-linkers (usually ECH) and special manipulations, namely freeze/thaw cycles, thermal treatment, supercritical CO_2_ drying and others [101,102]. These complicate industrial production of the hydrogels via the dissolution of cellulose in NaOH systems. Organic and mixed solvents, such as N-methylmorpholine-N-oxide (NMMO), dimethyl sulfoxide (DMSO)/LiCl, and N,N-dimethylacetamide (DMAc)/LiCl, have a moderate price, and the methods of dissolution usually do not require additional treatments except for heating. From this point of view, organic solvents seem to be fine candidates for the industrial production of the hydrogels. 

**Figure 4:**
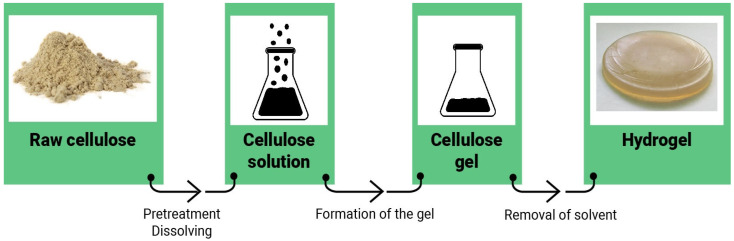
Schematic process of the cellulose hydrogel production from an organic or mixed solvent.

On the other hand, the solubility of cellulose in NMMO, DMSO/LiCl and DMAc/LiCl depend on many factors. Additionally, some organic solvents are inferior to others in terms of environmental friendliness. We will sum up the recent studies on the preparation of hydrogels from native cellulose to address these questions. The principal scheme of hydrogel production via dissolution is presented in Figure 4.

### 4.1. N,N-Dimethylacetamide/LiCl 

DMAc/LiCl is a direct non-derivatizing aprotic solvent capable of dissolving cellulose without chemical modification mainly via the disruption of the cellulose hydrogen-bonding network [38]. The solvent system DMAc/LiCl has been implemented for hydrogel production from cellulose of various origins. The reported sources of cellulose were conventional plant-derived, namely cotton, hardwood pulp, and more sustainable short-length flax fibers [86,103], bamboo [104,105], rice straw, sugarcane bagasse, thanaka heartwood [85], wheat husk [106], waste paper and cardboard [93].

The first step towards cellulose hydrogel is the treatment of raw material as it was discussed above. The majority of studies included multiple-step procedures for cellulose fibers, such as acid and/or alkali treatment, and bleaching [87,105]. However, in some cases, cellulose-containing wastes were used without preliminary cellulose extraction, but the dissolving ability in these cases was approximately 21–55% depending on the initial material [95].

The second non-compulsory stage of the production of hydrogels is the activation of cellulose. It is usually performed by solvent exchange, more specifically, by swelling in hot water, then rinsing with alcohol, and, after that, with DMAc [86,104,107]. Activation causes structural and morphological changes in cellulose, creating a favorable environment for dissolution and facilitating DMAc solvent diffusion into cellulose macromolecules [108].

The third dissolution step is carried out under constant stirring in a DMAc/LiCl system, where the concentration of the salt is 6–8 wt.%. It usually occurs under heating; however, there are strategies of dissolution at room temperature [86]. Important to note that both DMAc and LiCl must be dehydrated since the presence of water decreases the effectiveness of the solvent [109]. Dissolution results in a clear or semi-clear viscous solution with the concentration of cellulose from 0.5 to 5.0 wt.%. 

The dissolving ability of cellulose in DMAc/LiCl is reported to be 94.8–98.7 wt.% [86] and depends on the type of initial material, precisely, such factors as the degree of polymerization (DP), the impurity contents, supramolecular structure and crystallinity of cellulose [103]. The lower the crystallinity is, the higher the water retention capacity is. The amorphous regions of cellulose are expected to swell and accommodate water. Absorption in the amorphous regions of cellulose presumably involves strongly bound water (“hard to remove water”) [110]. 

The resulting cellulose solution may be transparent or colored in yellow in the case of lignocellulose, or green, yellow or brown when the waste paper was dissolved directly [95]. In [106], the solutions were additionally undergone a UV irradiation (353 nm) to improve mechanical performance.

The next step is the gelation of the cellulose solution. Gelation was defined by Hubbe as the inter-connecting of the macromolecular chains in some manner, such that essentially the whole structure becomes linked together [110]. Ishii assumed that the molecular aggregates can form the gel because of the large-scale fluctuation of the molecular chain density in the solution [111]. Gelation is accompanied by the appearance and strengthening of a spatial network between cellulose macromolecules in the solution of DMAc/LiCl [86]. Thus, LiCl splits into ions because of the formation of a new strong hydrogen bond between the hydroxyl protons of cellulose and the Cl^−^ ions of the salt [112], while Li^+^ cations are solvated by free DMAc molecules. This leads to the formation of a stable network of cellulose macromolecules and molecules of solvent. 

Gelation of the cellulose solutions in DMAc/LiCl occurs by spontaneous self-assembly and aggregation of lignocellulose chains in ambient conditions with a humid atmosphere for a period from 12 h up to 7 days [86,95,106]. Cruz-Medina and co-authors claimed that moisture of the atmosphere plays the role of antisolvent to coagulate cellulose [102]. In the studies [87,104], ethanol was used as a coagulant for the cellulose solutions in DMAc/LiCl. Upon gelation, the solutions are completely absorbed and the swollen samples of the organogels are formed. 

Finally, the organogels are rinsed with cold and hot distilled water several times, in order to remove residues from the solvent system. Thus, the cellulose hydrogels are prepared. They are physically stable as long as needed. It is important that cellulose hydrogels must be stored in an aqueous or highly humid medium in order to maintain a constant balance between the water molecules bound in the network and in the surrounding space [106]. The described procedure of the hydrogel preparation using DMAc/LiCl is simple to perform, it requires neither chemicals except for the solvent components nor sophisticated equipment and excessive electricity expenses. Despite the fact that the method of dissolving cellulose in DMAc/LiCl is often called “green”, the solvent itself is not an environmentally friendly compound. According to WHMIS-2015 (Workplace Hazardous Materials Information System) issued by CNESST (Committee on Standards, Equity, Health and Safety at Work in Quebec) [113], DMAc is a combustible liquid toxic if inhaled, that may cause drowsiness or dizziness, serious eye irritation, and damage to organs through prolonged or repeated exposure. Moreover, it is suspected of causing cancer and damaging fertility or the unborn child. Therefore, strict precautions must be taken when working with this solvent. In order to increase the environmental friendliness of solvents, it is necessary to use methods of regeneration from effluents, purification and reuse. From this perspective, DMAc meets the principles of environmental friendliness due to its recyclability [114]. 

### 4.2. Dimethyl Sulfoxide/LiCl

One more aprotic complex cellulose solvent is DMSO/LiCl, although it is not as popular as DMAc/LiCl. A group of Chinese researchers has been developing a method of fabrication of cellulose hydrogels and aerogels by the dissolution-regeneration process using the DMSO/LiCl solvent system. It was shown that DMSO/LiCl effectively dissolves cellulose or lignocellulose [93,115,116]. 

The procedure of the hydrogel preparation, in general, is similar for DMAc/LiCl and DMSO/LiCl. Dissolution of lignocellulose in DMSO/LiCl (concentration of LiCl is 8 wt.%) requires pre-treatment, e.g., ball-milling or ethylenediamine (EDA) complexation. While ball-milling causes a decrease in DP, EDA pre-treatment can avoid mechanical degradation [117]. According to Xia et al. [115], the dissolution of 2% pulp takes 24 h at room temperature and 5 h at 65 °C under stirring to obtain a homogeneous solution. Then, the solution is immersed in ethanol for gelation. The gel is thoroughly washed with ethanol, and then with water to remove LiCl, DMSO, and EDA. 

Recently, Xia and co-authors enhanced the method by applying the semi-interpenetrating polymer network (SIPN) strategy. After the solution of lignocellulose in DMSO/LiCl is prepared, N-isopropylacrylamide with a cross-linker and an initiator are added, and the mixture is stirred under heating until the total dissolution. In the next step, the solution gelates at 70 °C for 12 h [118]. Application of the SIPN strategy allowed externally added lignin to be successfully dissolved. As a result, the composite hydrogels obtain better mechanical strength and other properties. Another research group applied the same solution of lignocellulose in DMSO/LiCl crosslinking by tetraethyl orthosilicate in non-aqueous medium under catalysis with EDA to improve the properties of the lignocellulosic hydrogels [119].

In spite of many common features in the preparation of hydrogels from organic solvents with lithium chloride, the methods using DMSO/LiCl seem to be more complicated since it requires an additional step, namely ball-milling, complexation, and cross-linking of lignocellulose. However, usually, hydrogels obtained by the crosslinking have advantages in mechanical properties over non-crosslinked hydrogels. Additionally, from the ecological point of view, DMSO is preferable than DMAc, since it is non-toxic though combustible liquid [120]. Despite these advantages, the method of hydrogel preparation via dissolution in DMSO/LiCl has a very limited distribution. 

### 4.3. N-Methylmorpholine-N-oxide

NMMO is a well-known industrial solvent for the production of cellulose dissolved fibers in large quantities. The pulp can be dissolved easily without pre-treatment in NMMO for producing regenerated cellulosic films, food casings, membranes, sponges, beads, and other products [121]. Zhou and co-workers have successfully manufactured hydrogels via the dissolution of non-derivatized cellulose in NMMO in a vacuum oven set at a temperature of 110 °C and a vacuum of −0.1 MPa. The resulting organogels with cellulose concentration of 4–8% were rinsed in deionized water to change the solvent into water, as a result, cellulose hydrogels were obtained. The hydrogels were able to reswell in water after freeze-drying, with the water uptake rate and equilibrium swelling ratio increasing depending on the content of cellulose due to the increase in the number of hydrophilic hydroxyl groups [122]. Thus, for the higher reswelling ability of hydrogels, it is desirable to raise the cellulose content in the hydrogel, but the cellulose solubility limit is approximately 35% in NMMO [8].

The advantages of NMMO are the possibility of recovery, non-flammability, and relatively low toxicity. However, long-term exposure may result in disease of the airways, also this chemical accumulates in the human body [123]. From the industrial point of view, the problem is the high temperatures required for the dissolution which cause additional electricity expenses and increase overall costs. 

### 4.4. Alkaline Aqueous Systems

Neither lignocellulose nor pristine cellulose can be dissolved in water; but NaOH-based aqueous systems can lead to cellulose and lignocellulose dissolution due to the breaking of multiple intermolecular hydrogen bonds of polysaccharides by NaOH. The optimum conditions reported by Isogai and Atalla, involved swelling cellulose in 8–9 wt.% NaOH and then freezing it at −20 °C, followed by thawing the frozen mass at room temperature and diluting it with water to 5% NaOH. This protocol provided the complete dissolution of microcrystalline cellulose, essential for regenerated cellulose; however, the original cellulose, its mercerized form, and cellulose III samples had limited solubility values of only 26–37% [124]. Later, the freezing temperatures increased up to 0 °C, but it was shown that cellulose solubility is limited to celluloses of relatively low DP (<250) and crystallinity [38]. 

The addition of organic compounds such as urea or thiourea to NaOH solution (e.g., NaOH/urea/thiourea at 8/8/6.5 ratios) improve breaking of the hydrogen bonding of cellulose and prevents the approach toward each other of the cellulose molecules, leading to the perfect dispersion of cellulose to form a solution [65]. Urea and thiourea also help to prevent the reassociation of cellulose chains to increase the stability of the solution [38]. One more well-known method to improve the solubility in NaOH-aqueous systems is the pre-treatment of cellulose, e.g., mechanical (hydrothermal treatment and steam explosion), chemical (ethanol or hydrochloric acid), and enzymatic (cellulase enzyme) [38].

These findings became the basis of the procedure for the production of cellulose hydrogels. Unlike the organic solvents, alkaline aqueous systems make it possible to obtain a metallogel directly by a one-pot principle mixing the cellulose solution in NaOH-aqueous system with aqueous solutions of a metal salt as it was shown in Section 2 of this review. This significantly expands the boundaries of the use of hydrogels and simplifies the procedure for obtaining composite hydrogels. Thus, Song et al. obtained hydrogels from bacterial cellulose in NaOH/urea/water system by adding polyvinyl alcohol and AgNO_3_, the same as Lin et al. added chloroauric acid directly in the reaction flask where dissolution occurred [125,126]. To improve the mechanical properties of the hydrogels ECH can be added as a chemical crosslinker. In study [88], it was reported that the optimal molar ratio of ECH to the anhydroglucose unit of cellulose, which provided the superior mechanical properties of the hydrogel, was 0.7 in the range of 0.5–0.9. After crosslinking (if it was carried out), the solution of cellulose in NaOH was poured into the molds and cooled at the low temperature <4 °C [88,125,127]. Then, the hydrogels were rinsed in water until the complete removal of impurities.

The preparation of the hydrogels in alkaline water is considered an eco-friendly process first of all because of the absence of organic solvents. Even though the organic solvents are regeneratable and some of them are non-toxic, aqueous wastewater is way easier to recover. For cellulose dissolution in alkaline aqueous systems low concentrations (7–10 wt.%) of NaOH are used, therefore such harmful properties of sodium hydroxide as irritating and corrosive, may not be considered. Thus, an advantage of the alkaline water solvent is its low price and environmental friendliness, but such limitations as moderate solubility (only at certain DP and crystallinity) and stability of solutions, the need to use low temperatures and additional cross-linking stages should be considered.

Comparative analysis of the methods and conditions for the production of hydrogels from native cellulose, lignocellulose, or cellulose-containing wastes carried out in this chapter and summarized in Table 2, allows us to draw the following conclusions. It should be noted that in this analysis we did not consider cellulose derivatives as starting materials.

(1) Protocols for the production of hydrogels from cellulose are very similar for the three organic solvents considered, namely DMAc/LiCl, DMSO/LiCl, NNMO, while cellulose hydrogels obtained from NaOH-aqueous solutions involve cooling of the system.
(2) The most common solvents are DMAc/LiCl and NaOH; NNMO and DMSO/LiClare used less frequently.
(3) Each solvent has pros and cons (Table 2). Thus, the NaOH-aqueous system is inexpensive and environmentally beneficial; however, there are limitations to the dissolution of some types of cellulose. These drawbacks can be minimized due to extensive pre-treatment of cellulose which is an additional step in the production process. Usually, the hydrogels are synthesized from NaOH-solutions with such additives as urea and/or thiourea and crosslinking with ECH.

**Table 2:** Characteristics of processes for the production of cellulose hydrogels using organic solvents.

Solvent	Pre-Treatment of Cellulose	Dissolution Process	Gelation Process	Price *	Toxicity
DMAc/LiCl	solvent exchange (not compulsory)	25–80 °C	humid ambient condition	161 GBP/L (+28 GBP/100 g for LiCl)	harmful, flammable
DMSO/LiCl	ball-milling complexation	65–70 °C	ethanol, cross-linking	89 GBP/L (+28 GBP/100 g for LiCl)	non-toxic, flammable
NMMO	-	110 °C, −0.1 MPa	110 °C, −0.1 MPa	1110 GBP/kg	low toxic, non- flammable
NaOH/water	hydrothermal, steam explosion, chemical, or enzymatic treatment	(−20)–0 °C, addition of urea/ thiourea	cross-linking (not compulsory), cooling (approximately 0 °C)	35 GBP/kg	non-toxic, non- flammable

* The price is given in this study only for the purpose of an approximate comparison of reagents with each other, available online: www.fishersci.co.uk, accessed on 29 January 2023.

NMMO does not require pre-treatment of cellulose and it is environmentally friendly, but it has a high cost and requires high temperatures. DMSO/LiCl is indeed a green solvent, its environmental performance is very desirable, and its cost is the most attractive among the organic solvents, but it requires pre-treatment of cellulose-containing raw materials. DMAc/LiCl is the least environmentally attractive solvent because it has high toxicity, but the simple conditions for obtaining hydrogels make its industrial application easy. The reason why DMAc/LiCl is the most common solvent for cellulose hydrogel production lies precisely in the availability of the technique. 

## 5. Conclusions

Metallogels are obtained from ready-made cellulose hydrogels using the diffusion-reduction method, with the help of chemical and phyto-chemical reducing agents or without reducing agents due to the special properties of cellulose. This method makes it easy and inexpensive to obtain metallogels with transition metals. The second chemical method for the preparation of cellulose metallogels is associated with the ability of certain metal ions to initiate gelation and also act as cross-linking agents for cellulose. The second method, despite its attractiveness, has a limitation since it is suitable only for aqueous cellulose solvents.

Thinking outside the box and exploring new and unusual applications of cellulose-based metallogels may provide for a quicker adoption of their technology in a world that is becoming greener and more sustainable. In the first part of this review, we have presented that cellulose-based metallogels form an emerging class of biomaterials that can be produced from a broad variety of possible cellulosic raw materials, including non-conventional sources such as agricultural and food wastes as well as waste paper. 

Appropriate use of solvents and preparation methods offers promising opportunities to manufacture metallogels with tailored properties for a multitude of applications in many different domains, industries and environmental areas of application: textile, agriculture, horticulture, personal hygiene products, biomedical, pharmaceuticals, etc. The in-depth literature review has revealed that DMAc/LiCl and NaOH-aqueous systems are the most commonly used solvents for the production of cellulose hydrogels. NaOH-aqueous system is inexpensive and environmentally beneficial; however, it requires special pretreatment and conditions as well as crosslinking with ECH. DMAc/LiCl is the least environmentally attractive solvent, but the process of manufacturing hydrogels is simple and attractive from the industrial point of view. Last, but not least, a comprehensive overview of the main methods used for hydrogels preparation, having in mind that is essential to improve the industrial technology to produce the desired products in a cost-effective approach, was presented. 

## Figures and Tables

**Table 1:** Cellulose content in various non-wood sources.

Raw Material	Cellulose Content, %	Reference
rapeseed stalks	38–44	[78,80]
corn stalks	35–42	[82]
wheat straw	29–51	[80]
thanaka heartwood	28.5	[87]
tea leaf	22	[89]
rice straw	21.6–48	[80,87]
sugarcane bagasse	20.3–48	[80,87]
okara	8.8	[88]
waste paper	82–95	[99]
